# Olfactory Dysfunction in COVID-19 Patients in a Tertiary Care Hospital; A Cross-Sectional Observational Study

**DOI:** 10.22038/IJORL.2024.76275.3557

**Published:** 2024-05

**Authors:** Sreedevi Nunkappa Thippeswamy, Kamalesh Thagadur Nataraju

**Affiliations:** 1 *Department of ENT, JJM Medical College, Davanagere, Karnataka, India.*; 2 *Department of General Medicine, SS Institute of Medical Sciences and Research Centre, Davanagere, Karnataka, India.*

**Keywords:** Anosmia, Coronavirus, Smell.

## Abstract

**Introduction:**

Sudden onset of olfactory dysfunction (OD) manifesting as hyposmia and/or anosmia occurred in many COVID-19 patients, with a frequency as high as 85.6%. Given the morbidity and mortality of COVID-19, it is important to recognize the symptoms early so that the infected person can be diagnosed, isolated and treated early. Hence, this study was undertaken to know the prevalence of Sino-nasal symptoms with special reference to olfactory dysfunction in COVID-19 patients.

**Materials and Methods:**

It is a cross sectional observational study involving 160 COVID-19 patients aged 18 to 100 years selected by universal sampling. OD was analyzed and compared with various inflammatory markers and Sino-nasal symptoms. Patients were followed up until their discharge from the hospital or until death due to COVID-19 related health issues.

**Results:**

Out of 160 subjects included in the study, 61.88 % of the study participants were males and 38.13% were females. The mean age was 44.50 ± 16.43 years. A total of 51 patients (31.87%) developed OD. Fifty one (31.87%) patients developed OD (anosmia/hyposmia). Among the individuals with anosmia/hyposmia, majority of patients (n=26) (50.98%) complained of more than 75% loss of smell sensation. Mean duration of anosmia/hyposmia was 9.92 ± 3.71 days. OD correlated with serum ferritin levels (p=0.0453).

**Conclusion:**

Anosmia/hyposmia was found in significant proportion of patients with covid-19 which correlated with the disease severity and serum ferritin levels and hence can serve as surrogate marker of disease severity.

## Introduction

Coronavirus disease 2019 (COVID-19) is caused by novel coronavirus which is now referred to as severe acute respiratory syndrome coronavirus 2 (SARS-CoV-2) (1). The disease was first detected in December 2019 in Wuhan seafood market, China. SARS-CoV-2 is highly infectious (2). It has a reproduction number (R0) of 2 to 3, which means that an infected individual can transmit the infection to 2 to 3 individuals (2). This is the reason for the pandemic leading to millions of individuals being infected and has killed lakhs of people across the globe. COVID-19 is characterized by fever, cough and breathlessness along with constitutional symptoms like fever and myalgia (3-5). Nasal cavity, which is the most common site of viral entry is the seat of vigorous viral replication (6). Nasal epithelium contains angiotensin converting enzyme 2 (ACE2) receptor which acts as a receptor for viral entry (7). In-spite of this fact, nasal symptoms are reported by less than 10% of infected patients (8,9). The reason for this paucity of symptoms is not known. Over a period of time, it was observed that sudden onset of olfactory dysfunction (OD) manifesting as hyposmia and/ or anosmia occurred in many COVID-19 patients, with a prevalence as high as 85.6% (10-12). Interestingly, patients with anosmia did not have accompanying nasal obstruction or other rhinitis symptoms (10). Therefore, insult to olfactory receptors by the virus is the likely logical explanation of the mechanism of OD. This mechanism has also been proposed earlier for other upper respiratory viral infections (13). Understanding the mechanism of sensorineural olfactory loss may provide novel information on the pathogenesis of novel coronavirus. 

The olfactory epithelium which lines the roof of the nasal cavity contains olfactory neurons which are in direct contact with the environment (6). 

The total surface area of the nasal cavity is 150 cm2, of which 9 cm2 is formed by the olfactory epithelium (14). The olfactory epithelium which contains ACE2 receptors may act as the portal of viral entry into central nervous system leading to encephalitis. This has been confirmed by intranasal inoculation of SARS-CoV-1 in mice (15,16). The time taken to recover from anosmia or hyposmia is not very clear at this point of time. It is believed that it may follow the pattern of other post viral syndrome (17). It is expected that the complete recovery may take several months (18).

*Need For the Study*

Given the morbidity and mortality of COVID-19, it is important to recognize the symptoms early so that the infected person can be diagnosed, isolated and treated early. Symptomatology of COVID-19 varies from time to time and place to places across the globe. Many studies have variable results and lack comparison of olfactory symptoms with inflammatory markers in COVID-19. 

Hence, this study was undertaken to know the frequency of Sino-nasal symptoms with special reference to olfactory dysfunction in patients admitted to KVG medical college and hospital. Also, we intended to find the relationship between the inflammatory markers and the occurrence of OD in COVID-19. These symptoms may serve not only as marker of COVID-19 (especially when there is paucity of symptoms) but also act as indicator of progression to severe COVID. 

*Objectives*

Primary objectives:

- To find the frequency of Sino-nasal symptoms in patients with COVID-19
- To find the frequency of olfactory dysfunction in patients with COVID-19

Secondary objectives:

- To find the relationship between classical symptoms of upper respiratory viral infection (rhinorrhea, nose block and headache) with the occurrence of olfactory dysfunction.

- To find the relationship between olfactory dysfunction and disease progression in COVID-19.

## Materials and Methods

This was a cross-sectional study involving all patients with COVID-19 admitted in KVG Medical College and Hospital, Sullia. The diagnosis of COVID-19 was made by either of the following

- Presence of SARS-CoV-2 RNA detected by reverse transcription polymerase chain reaction (RT-PCR) in the nasopharyngeal or oropharyngeal swab specimens.
- Positive Rapid Antigen Test for SARS-CoV-2.

Any new changes in the diagnostic criteria by the local (Department of health and family welfare, Government of Karnataka) or national (Ministry of health and family welfare, Government of India) COVID-19 interim guidelines was implemented as and when the guidelines were updated. Patients with COVID-19 were classified based on the severity into 3 categories as per Indian Council of Medical Research (ICMR) (Table .1) (19).

**Table 1:** Indian Council of Medical Research (ICMR) classification (19) of COVID-19 severity

**Category **	**Criteria**
Group A	Asymptomatic/patients with mild symptoms Respiratory rate less than 24 per minute and SpO2 more than 94% in room air.
Group B	Symptomatic patient with mild to moderate pneumonia with no signs of severe disease Respiratory rate 24 to 30 per minute and SpO2 90% - 94% in room air.
Group C	Symptomatic patient with severe pneumonia with any of the following Respiratory rate more than 30 per minute SpO2 less than 90% in room air or less than 94% with oxygen Acute respiratory distress syndrome (ARDS) Septic shock

All patients were investigated and treated as per local (Department of health and family welfare, Government of Karnataka) or national (Ministry of health and family welfare, Government of India) COVID-19 treatment protocol. 

All patients admitted were screened for Sino-nasal symptoms and olfactory dysfunction at the time of admission after obtaining the written informed consent. The duration of each symptom was recorded. Patients were followed up until their discharge from the hospital or until death due to COVID-19 related health issues. The following data were recorded during the follow up.

- Improvement or worsening of the above-mentioned symptoms
- Time taken for partial or complete recovery from OD

Study design: Cross-sectional study

Study population: Patients with COVID-19 admitted to KVG medical college and hospital.

Study period: 25/08/2020 to 25/11/2020

Sample size: All patients with COVID-19 admitted during the study period were included.

Sampling technique: Universal sampling

Interventions involved in the study: None

Drug trials involved: None

Institutional ethical committee clearance was obtained. 

***Inclusion criteria:***

 All patients with COVID-19 aged between 18 to 100 years admitted to KVG medical college and hospital.

***Exclusion criteria:***

- Patients with pre-existing olfactory abnormality (due to any etiology)
- Patients who had past history of atrophic rhinitis
- Patients who had anterior cranial fossa fracture/head trauma/severe nasal injury

***Assessment of Sino-nasal symptoms:***

- Running nose/nasal discharge
- Nose block
- Headache/facial pain
- Excessive sneezing
- Nasal itching
- Post-nasal drip

***Assessment of olfactory dysfunction (OD):***

 Measured by subjective reporting by patients. Any degree of reduced small sensation was considered as hyposmia and similarly any degree of increased smell sensation is considered as hyperosmia.

- Anosmia: absence of sense of smell
- Hyposmia: reduced sense of smell
- Hyperosmia: increased sense of smell
- Parosmia: alteration in the quality of smell

**Following data were collected:**

1. Demographic details: Age, gender
2. Comorbid medical conditions
3. Symptoms related to COVID-19
4. Olfactory symptoms (as mentioned above)
5. Investigations - Haematological parameters like Complete blood count, Renal function tests (serum creatinine, blood urea), liver function tests (serum bilirubin, serum albumin, SGOT, SGPT), blood sugars [Random blood sugar (RBS), glycated haemoglobin percent (HbA1c)], inflammatory markers [C-reactive protein (CRP), serum ferritin, Lactate dehydrogenase (LDH), D-dimer].

***Materials and Methods***

The data was entered in Microsoft office Excel 2007 and SPSS version 21 was used for analysis of data. The frequency of Sino-nasal symptoms and olfactory dysfunction was calculated. The relationship of these symptoms was correlated with the disease inflammatory markers. The data was shown in the form of percentages and means. Chi-square test & t test was used for analysis. p value of less than 0.05 is taken as statistically significant. Shapiro wilk test was used to assess the normality. 

## Results

This cross-sectional study included 160 patients diagnosed with COVID-19. In this time bound study, 61.88 % of the study participants were male and 38.13 % were females (Table.2).

 Majority of the participants were in the age group between 20 and 60 years. 

The mean age of male study participants was 44.96 ± 16.28 years, and that of female study participants was 43.75 ± 16.78 years. The mean age of the whole study population was 44.50 ± 16.43 years.

**Table 2:** Age and gender distribution of the study population

**SNO.**	**Age Distribution**	**Male NO. (%)**	**Female NO. (%)**
1	18 – 19 Years	3 (1.87%)	0 (0%)
2	20 – 29 Years	15 (9.37%)	19 (11.87%)
3	30 – 39 Years	25 (15.62%)	9 (5.62%)
4	40 – 49 Years	17 (10.62%)	11 (6.87%)
5	50 – 59 Years	20 (12.5%)	10 (6.25%)
6	60 – 69 Years	11 (6.87%)	6 (3.75%)
7	70 – 79 Years	6 (3.75%)	5 (3.12%)
8	80 – 89 Years	1 (0.62%)	1 (0.62%)
9	90 – 99 Years	1 (0.62%)	0 (0%

**Fig 1:**
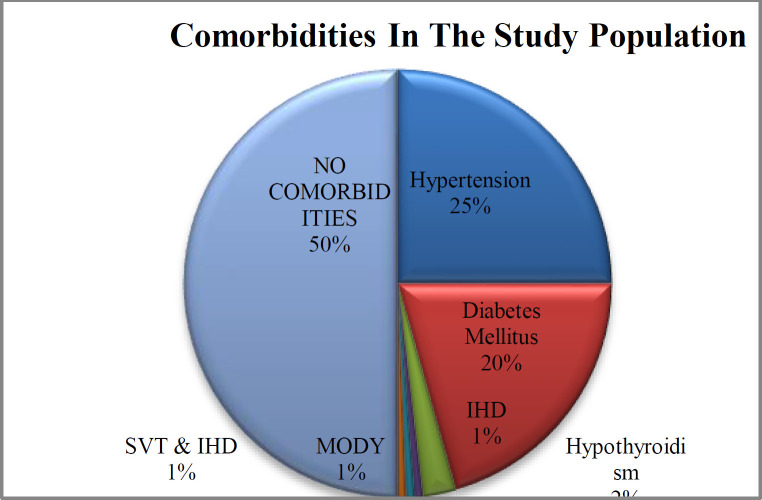
Distribution of medical comorbidities among study population

Hypertension and Diabetes mellitus were the most common comorbid conditions in the study accounting for 25% and 20.62% respectively. 80 (50%) of the study participants did not have any identifiable comorbid conditions (Figure 1). 

In this study we categorized the Covid-19 patients in 3 disease severity category as per ICMR protocol (category A, B, C). 

This categorization is based on the in-hospital follow up. The participants who were initially in category A and later progressed to category B were included in category B for the analysis. We, observed that majority of the patients (n=107) belonged to category B, accounting for 66.87%. Forty-three (26.87%) and ten (6.25%) of patients belonged to category A and C respectively. 

The mean vitamin D3 levels in the study was 16.38 ± 9.12 ng/mL which is the deficiency range. The mean CRP level was 19.31 ± 43.88 mg/L which is more than the normal upper limit (10mg/L). The mean LDH level was 406.42 ± 127.55 IU/L which is also beyond the normal range (105-333 IU/L). The mean D-dimer level was 0.59 ± 1.03 mg/L which is slightly above the normal upper limit (0.5 mg/L). These observations suggest that there is significant inflammation (p value for CRP = 0.8081, p value for LDH = 0.2744, p value for D-dimer = 0.1043) in patients with Covid-19 across all categories of severity. 

In this study, we collected serial values of CRP, serum ferritin levels, serum LDH 1 levels and D-dimer levels (which are done as per protocol of the ministry of health and family welfare). At least 3 values of the above mentioned parameters were collected. It was observed that there was improvement in the serial CRP levels as compared to the gradual worsening of the D-dimer and serum ferritin levels. However, there was no appreciable change (p value = 0.2744) in the serial LDH 1 levels (Table 3). The frequency of various Sino-nasal symptoms (other than olfactory dysfunction) is mentioned in Table 4.

**Table 3:** Serial serum inflammatory markers

**SNO.**	**PARAMETERS**		
1	CRP 1 (mg/L)	CRP 2 (mg/L)	CRP 3 (mg/L)
	19.31 ± 43.88	17.42 ± 55.95	15.48 ± 51.55
2	FERRITIN 1 (mcg/L)	FERRITIN 2 (mcg/L)	FERRITIN 3 (mcg/L)
	191.51 ± 234.03	228.10 ± 241.27	216.21 ± 220.80
3	LDH 1 (IU/L)	LDH 2 (IU/L)	LDH 3 (IU/L)
	406.42 ± 127.55	394.52 ± 130.25	424.94 ± 130.11
4	D DIMER 1 (mg/L)	D DIMER 2 (mg/L)	D DIMER 3 (mg/L)
	0.59 ± 1.03	0.61 ± 0.63	0.68 ± 1.09

**Table 4:** Frequency of Sino-nasal symptoms (excluding smell abnormality) in the study population

**SNO.**	**SYMPTOMS**	**NO. OF PATIENTS** **NO. (%)**	**Mean duration** **(days)**
1	Nose Block	5 (3.12%)	9.4 ± 5.94
2	Running Nose	13 (8.12%)	5.69 ± 2.56
3	Excessive Sneezing	0 (0%)	0
4	Nasal Itching	0 (0%)	0
5	Epistaxis	0 (0%)	0
6	Post Nasal Drip	0 (0%)	0
7	Headache	23 (14.37%)	7 ± 3.68
8	Facial Pain	0 (0%)	0

A total of 51 patients (31.87%) developed olfactory dysfunction. 10% of the patients having symptoms of classical upper respiratory infection (URI) developed olfactory dysfunction. There was significant relationship (p=0.045) between presence of URI and development of olfactory dysfunction. Fifty one (31.87%) patients developed olfactory dysfunction in the form of anosmia or hyposmia (Table 5). 

**Table 5:** Olfactory dysfunction (OD) in the study population

**Sl. No**	**Symptoms**	**No. of Patients (%)**
1	Anosmia / Hyposmia	51 (31.87%)
2	Hyperosmia	0 (0%)

Among the individuals with anosmia/ hyposmia, majority of patients (n=26) (50.98%) complained of more than 75% loss of smell sensation followed by 50-75% loss of smell sensation in twenty two (43.13%) patients. Mean duration of anosmia/hyposmia among 51 patients was 9.92 ± 3.71 days (Table 6). We compared the inflammatory markers like CRP, serum ferritin, serum LDH-1 and D-dimer in patients with and without smell dysfunction across all three-disease category (category A, B & C) (Table 7and 8). 

**Table 6:** Severity of Olfactory dysfunction (OD)

**SI.**	**Severity Of Symptoms** **(% of Loss of Smell Sensation)**	**Anosmia/Hyposmia** **No. (%)**	**Mean Duration of Symptoms** **(Days)**
1	< 25 %	3 (5.88%)	9 ± 1
2	25 – 50%	0 (0%)	0
3	50 – 75 %	22 (43.13%)	9.59 ± 3.69
4	> 75%	26 (50.98%)	10.30 ± 3.96

**Table 7:** Mean serum inflammatory markers in COVID patients with olfactory dysfunction

**Olfactory Dysfunction**	**Mean CRP** **(mg/l)**	**Mean Ferritin** **(mcg/l)**	**Mean LDH-1** **(IU/L)**	**Mean D Dimer** **(mg/l)**
Olfactory Dysfunction	21.35 ± 50.69	245.58 ± 303.01	434.82 ± 120.22	0.41 ± 0.32
No Olfactory Dysfunction	18.35 ± 40.52	166.21 ± 190.00	393.13 ± 129.24	0.68 ± 1.22
T Value	0.4016	2.0182	1.9432	1.5732
p Value	0.6885	0.0453*	0.0538	0.1177

(statistical method: student t test)

CRP: C-Reactive Protein (Normal blood CRP level: <0.3mg/dl) LDH-1: Lactate Dehydrogenase-1 (Normal serum LDH-1 level: 105 to 233 IU/L). Normal serum ferritin level: 12 to 150 ng/ml. Normal D-dimer level: <0.5mg/L. Statistical test used for Table 7 is student t test.

**Table 8:** Olfactory dysfunction in covid patients belonging to different categories

**Olfactory Dysfunction**	**Mean CRP** **(mg/l)**	**Mean Ferritin** **(mcg/l)**	**Mean LDH** **(IU/L)**	**Mean D Dimer** **(mg/l)**
Category A				
Olfactory Dysfunction	25.36 ± 81.95	159.38± 137.87	438.50 ± 104.73	0.29 ± 0.20
No Olfactory Dysfunction	14.51 ± 38.22	189.94 ± 238.49	386.74 ± 146.47	0.54 ± 1.45
T Value	0.5963	0.4434	1.1811	0.6475
p Value	0.5543	0.6598	0.2444	0.5209
Category B				
Olfactory Dysfunction	20.48 ± 34.49	234.43 ± 292.62	419.82 ± 115.28	0.47 ± 0.35
No Olfactory Dysfunction	14.66 ± 29.45	151.93 ± 167.75	390.54 ± 123.42	0.45 ± 0.44
T Value	0.9048	1.8516	1.1761	0.2038
p Value	0.3676	0.0669	0.2422	0.8389
Category C				
Olfactory Dysfunction	8.51 ± 11	1044.1 ± 220.47	671.5 ± 89.80	0.24 ± 0.14
No Olfactory Dysfunction	65.46 ± 89.19	208.69 ± 193.80	439.62 ± 121.12	3.29 ± 2.02
T Value	0.8625	5.3550	2.4927	2.0354
p Value	0.4135	0.0007*	0.0374*	0.0762

## Discussion

In a meta-analysis of 3563 patients with COVID-19 infection, it was found that the mean prevalence of self-reported loss of smell to be 47% (20). Moein *et al. *demonstrated that 98% of the hospitalized study participants (n=60) had some degree of OD on formal testing, whereas only 35% had self-reported loss of smell or taste. Hence, formal testing for OD is more accurate than just relying on self-reporting of the symptoms (21). Loss of smell may be the only presenting feature for patients with COVID-19 (12). OD may be preceding other symptoms in 20% (20).

Yan et al. observed that OD was associated with milder COVID-19 disease and only 26.9% of the hospitalized COVID-19 patients reported anosmia/hyposmia (22). 

In contrast to this our study revealed higher inflammatory markers in patients with OD compared to those without OD. One of the probable reasons for this difference in observation is that, in severe COVID-19 disease the focus is on respiratory distress and loss of appetite and hence OD may be overlooked by the patients as well as the treating physicians. Vaira LA et al. observed no significant correlation between OD and the disease severity (23).

In a study conducted by Klopfenstein T et al. 47% of 114 patients had OD and among them less than 5% had running nose or nose block which are typically associated with common cold (24). In our study, 45.71% of the patients with OD had nasal symptoms of rhinitis. This high percentage is because we included even those with milder COVID-19 disease. Even though the OD resolves on its own, in some patients it may take months to resolve. Whether this OD remains permanent or lasts longer is still not known. Hence, OD cannot be left unattended. It has been found that olfactory training in the form of repeat and deliberate sniffing of a set of odorants (commonly lemon, rose, cloves, and eucalyptus) for 20 seconds each at least twice a day for at least 3 months (or longer if possible) is beneficial in faster recovery of smell (23,25). 

Nasal steroid sprays, intranasal vitamin A and supplements of alpha lipoic acid and omega 3 fatty acids have been tried with a marginal benefit (26). In a metanalysis, it was found that 74.1% of participants their sense of smell at 30 days, 85.8% at 60 days, 90.0% at 90 days, and 95.7% at 180 days, with a median recovery time of 14.9 days (27). In our study, mean duration of anosmia/hyposmia among 51 patients was 9.92 ± 3.71 days. We have not followed up the patients until complete recovery of olfaction. Hence, this is one of the limitation of the study. One of the implications of OD is the effect it has on the quality of life and nutrition. It has been found that many of the patients with persistent OD have depression and low self-esteem due to poor quality of life and vice-versa (28).

*Strengths of the study:*

1. This study compares Sino-nasal symptoms with inflammatory markers unlike other studies found in the literature.
2. This study also compares the frequency of Sino-nasal symptoms across all three disease category (category A, B & C).

*Limitations of the study:*

1. The assessment of OD was subjective. No objective method was included to detect minor OD.
2. Long term follow up of the patients is not done to know the average time taken for complete recovery from OD.
3. Effect of these symptoms on the quality of life and mental health is not evaluated.
4. The effect of medications on the occurrence and severity of OD is not evaluated.

*Take home message:*

COVID-19 pandemic has emerged and is re-emerging across various parts of the globe and has caused devastation in the health care system. The only way to contain the pandemic is to prevent, detect early and treat early. Olfactory dysfunction (especially anosmia) can be a useful symptom to detect COVID-19 patients early. 

## Conclusion

Anosmia (or hyposmia) was found in significant proportion of patients with covid-19 and their occurrence correlated with the disease severity and serum ferritin levels and hence can serve as surrogate marker of disease severity. These symptoms also contribute to significant proportion of symptoms in long Covid syndrome and thus contribute to growing burden of long covid. Therefore, it is important to recognize these symptoms early and address the problem to avoid its effect on the quality of life and mental health. 
